# Contribution of an Online Intervention to Developing Communities of Practice: Mixed Methods Evaluation of an Online Safety Hub to Address Harmful Online Content in Relation to Self-Harm and Suicide

**DOI:** 10.2196/72130

**Published:** 2026-01-14

**Authors:** Arne Mueller, Gemma Hughes, Gregory Maniatopoulos

**Affiliations:** 1 Faculty of Health Sciences, University of Hull, Kingston upon Hull, United Kingdom; 2 School of Business, University of Leicester, London Road, Leicester, LE21RQ, United Kingdom, +44 (0)116 252 5108

**Keywords:** communities of practice, digital intervention, online intervention, mental health professionals, self-harm, suicide, suicide prevention

## Abstract

**Background:**

Online harm affects many people and has been associated with self-harm and suicidal ideation. Although there is an emerging body of evidence that addressing adverse online experiences should be part of the support offered to people who are at risk of self-harm and suicide, there has been little guidance to date on how this support might be provided and how safe conversations can be had on the subject. A UK charity dedicated to offering emotional support to anyone experiencing mental discomfort, having difficulty coping, or being at risk of suicide developed a digital intervention, the Online Safety Hub (the Hub), to address this shortfall.

**Objective:**

The study aimed to evaluate the impact of the Hub on practitioners (people who provide support) and people with lived experiences of suicide and self-harm and to determine what learning environment is best suited to increase and maintain learning in the context of the Hub.

**Methods:**

A sequential explanatory mixed methods evaluation comprised a rapid literature review, data collected from people with lived experience (n=6) and practitioners through an analysis of the Hub’s activity data, 2 surveys (survey 1: n=45; survey 2: n=368), interviews (n=9), and focus groups (n=7). Surveys were analyzed for descriptive purposes only, and the interview and focus group analyses comprised coding of data and thematic analysis. The study design was informed by a panel of people with lived experience of online harm resulting in either self-harm and/or suicidal ideation.

**Results:**

Initially, the evaluation found limited uptake of the Hub. Engagement with the Hub was impeded by a lack of clarity on the part of practitioners as to whether they were the intended audience. The evaluation process prompted the charity to design and deliver webinars to facilitate uptake of the Hub. Practitioners who engaged with the Hub via webinars found the content useful and were able to consider incorporating their learning into practice. The webinars offered a more social learning experience than individual engagement with the Hub, providing a community of practice for people with common interests across diverse organizational settings. Opportunities for shared learning and the supportive nature of the community of practice were valued when learning about the sensitive and difficult topic of online harm in relation to self-harm and suicide. The Hub contributed to awareness-raising and shared learning.

**Conclusions:**

Online resources alone may not be sufficient for an intervention to effectively raise awareness and change practice. Social learning facilitated through communities of practice can enhance engagement, uptake, and learning.

## Introduction

### Background

When people are experiencing psychological, psychiatric, or emotional difficulties, the internet can have a pivotal function in managing their stress and emotions. For people with suicidal ideation or who self-harm, however, accessing the internet during periods of suicidal crisis or when experiencing the urge to self-harm does not always have positive and supportive effects [1,2]. The potential for harm to come to vulnerable individuals, particularly those who struggle with suicidal thoughts or self-harm, through their engagement with the internet is a growing concern. Not only does the internet expose individuals to suicide and self-harm–related content passively, for example, when they come across such content accidentally, but it can also provide them with tools they might actively seek out for self-harm and suicide-related purposes, which can lead to a downward spiral of increased suicidality and despair [3]. Therefore, the internet is an essential area of concern in terms of mental health, suicide, and self-harm, with the question of how to address engagement with online content increasingly central to the consideration of mental health support.

Online interventions such as training websites, e-learning modules, and webinars are standard means of contemporary professional development in many fields, including health and medicine [4,5]. Offered to staff starting new roles and as refresher training, online interventions allow organizations to provide mandatory and voluntary training efficiently and flexibly. Online interventions are available 24 hours a day, 7 days a week, allowing users to undertake training at their chosen time and pace, on site or off site, within a given timeframe [6]. Beyond mandatory staff training, online interventions are also used by professionals who seek to develop in particular areas needed for their roles and enhance their practice [7,8]. However, the availability of online resources does not necessarily result in uptake; the design, content, and format of the online resources play a role in adoption [9], and other less easily adjustable factors, such as workload pressures, can prevent uptake.

The Samaritans, a UK charity dedicated to offering emotional support to anyone experiencing mental discomfort, having difficulty coping, or being at risk of suicide, developed an online intervention in 2022 to raise awareness and increase knowledge about online harm: the Online Safety Hub (hereafter referred to as the Hub) [10]. The Hub was coproduced by the charity’s online excellence team and people with lived experience of online harm resulting in either self-harm and/or suicidal ideation. It comprised a range of resources aimed at supporting anyone involved in counseling people about suicide or self-harm who may have been exposed to online harm. The Hub is one response in the United Kingdom to concerns about harmful online content increasing risks of suicide and self-harm; other responses include legislation (the Online Safety Act 2023) and engagement with online providers. The latter was taken on by the Office of Communications, the designated online safety regulator in the United Kingdom, that set out detailed plans requiring providers of online services to protect their users from illegal content and to protect children from harmful content, including pornography. However, to the authors’ knowledge, there is currently no standard of care for practitioners in the United Kingdom when it comes to advice directed toward online harm. In addition, there is no published research about the implementation and use of online interventions such as the Hub by practitioners to address suicide and self-harm. The evaluation of the Hub provides an opportunity to address this knowledge gap. We analyzed the data gathered through the evaluation to answer the research question: how can diverse practitioners be supported by an online intervention to enhance their practice?

The concept of communities of practice (CoPs) can be used to account for how the online intervention connected diverse practitioners working in a wide range of organizational settings but with a common interest in supporting people at risk of suicide and self-harm. The term was coined by Lave and Wenger [11] in 1991 to describe groups of people who share an interest in something they do and learn how to do it better as they interact regularly [12]. These groups are found in various settings, such as educational institutions, professional networks, and business organizations. CoPs are characterized by a flat hierarchy and a confidential atmosphere, which promotes knowledge sharing and emotional support [13]. Although traditionally described as communities that regularly meet face to face, CoPs also exist online. Online CoPs have been found to manifest the characteristics of conventional CoPs, with members actively engaging in shared practice and identity development while pursuing a joint enterprise [14]. Online CoPs are further considered to offer greater accessibility and flexibility when compared to traditional CoPs; however, it is noted that they may face challenges in fostering trust among their members due to the more anonymous online format [15]. While traditional CoPs excel in building personal connections, they are at the same time constrained by physical and temporal limitations [16]. Furthermore, online CoPs can be a valuable interface for connecting experiences and uncovering challenges and opportunities in various fields, such as public health [17].

### An Overview of Online Harm

Online harm has been an increasing social and policy concern in the United Kingdom, leading to the Online Safety Act 2023. The act provides a regulatory framework that aims at “making the use of internet services… safer for individuals in the United Kingdom” [18]. By doing so, it regulates how social media and other internet services moderate and manage harmful and abusive online content to protect users from being exposed to certain harm. The legislation covers the restriction of dangerous content and the removal of illegal material. However, broader harms relating to online activity are still prevalent and are of particular concern in relation to suicide and self-harm.

There is no single definition available for online harm. Online harm is associated with harmful relationships mediated by online connections (eg, cyberbullying), interrelated with concerns about internet “addiction” or pathological use of the internet. Online harm can, therefore, mean any detriment arising from or through the use of the internet. It is also associated with specific content that presents and promotes self-harm and suicidal behavior. Such content can provide harmful information and has been understood by some researchers as acting as a form of “contagion” [19,20]. A contagion is perceived as a multiplicator mechanism resulting from the presence of suicidal content in media, including social media, that leads to people copying behavior they would otherwise not have been confronted with [20,21]. People might actively seek out content related to self-harm and suicide or inadvertently come across content that might be experienced as harmful. Such online interactions have the potential to adversely affect people in a range of ways, including causing some people to feel the urge to self-harm or ideate suicide [22]. “Online” in this context encompasses the full range of modes of connecting via the internet (including mobile devices, apps, and websites) and the modality of “being” online and participating in online “spaces.”

Online harm is predominantly associated with young people. However, anyone using the internet may be harmed by online activities, such as cyberbullying or cyberstalking, or targeted, for example, by online scams or, for women, online misogyny [23,24]. Previous research indicates that online harm can lead to adverse emotional and psychosocial consequences, particularly for those with personality traits such as sensation-seeking, low self-esteem, and psychological difficulties, as well as social factors like lack of parental support and peer norms [23,25]. Exposure to harm-advocating online content, such as proeating disorder, pro–self-harm, and prosuicide material, has been associated with lower subjective well-being among young people [26,27], with similar risks for vulnerable adults. Additionally, real-world online activity data have shown that multiple online risk factors, including cyberbullying, violence, hate speech, and sexual content, are associated with youth suicide-related behavior [28]. However, not all online risks result in self-reported harm, and the prevalence of these risks does not appear to rise substantially with increasing access to mobile and online technologies [24].

Harmful content alone will not necessarily lead to adverse outcomes such as increases in self-harm or suicide. The subjective nature of online harm is emphasized in several studies [29,30]. Content and interactions that might be experienced as harmful for one person at a point in time (eg, by normalizing or reinforcing self-harm) could be helpful for another person or, indeed, that same person at a different point in time if they encounter it as part of seeking help—and it is essential to note that some people prefer to seek help online rather than in person. People with mental health conditions, for example, often use the internet to learn about their condition, and research has further indicated that young adults with mental health conditions frequently use social networking sites, for example, as a means to find support and build a community around the issue [31]. Furthermore, adolescents often share self-injury content online, which can have both positive and negative effects [32]. Positive effects can include getting positive reactions from peers, such as being offered help, connecting, or receiving empathy, whereas negative effects can include eliciting negative online comments containing harassment and being misunderstood.

Efforts to avoid and address online harm include legislation, moderation of content and interactions (for example, by social media platforms such as Facebook, Instagram, and YouTube, as well as by individual moderators of sites and groups), media guidelines and frameworks aimed at producers of content and platforms, guidance for practitioners supporting people experiencing online harm (such as that offered by the Samaritans), and support for those directly experiencing online harm. People with mental health problems sometimes disclose their online experiences with their therapists [33]; however, scrutinizing the online activity of clients proactively has so far been a mostly neglected area in mental health practices. Nonetheless, empirical research indicates that mental health practitioners could helpfully address the online activity of young people during mental health consultations [34], although statistical evidence about the total rate of mental health practitioners who inquire about their patients’ online activities is not yet available. Accordingly, previous research identified a lack of guidance for practitioners on how to have those conversations [35].

In sum, the online environment is dual in nature; it can be both harmful and helpful. Certain content can be distressing and triggering, and online connections can be abusive and exploitative. However, people can find solace and help online through positive connections and useful information. Efforts to shut down online harm risk removing opportunities for online help. Resources to support people exposed to online harm and who are at risk of self-harm and suicide are therefore needed. This paper draws on an evaluation of an online intervention that aims to assess the impact of the Hub on practitioners (people who provide support) and people with lived experiences of suicide and self-harm and to determine what learning environment is best suited to increase and maintain learning in the context of the Hub.

## Methods

### Research Setting

The Hub consists of a public-facing website containing guidelines for practitioners, downloadable content, including handouts, and a social media tool kit. The website is supplemented by an e-learning module hosted on a National Health Service (NHS) platform, which requires a user account to access. The Hub and resources were co-designed with people with lived experience. The Hub was launched in November 2022 and advertised to a range of professional networks, including mental health practitioner networks, the Samaritans’ networks of practitioners and supporters, and on social media including LinkedIn, throughout the United Kingdom in early 2023, resulting in a considerable initial uptake among practitioners in subsequent months, with activity data indicating up to 350 module launches per month. The resources were primarily aimed at encouraging practitioners to raise the topic of online harm and to highlight the potential benefits of having supportive, safe conversations about online harm in the context of supporting people with self-harm and suicide.

The study aimed to evaluate the impact of the Hub on practitioners (people who provide support) and people with lived experiences of suicide and self-harm and to determine what learning environment is best suited to increase and maintain learning in the context of the Hub.

An evaluation of the Hub was conducted between 2023 and 2024 to assess practitioners’ engagement and experience with the Hub, its impact on their practice, and barriers and enablers to its implementation. In addition, the evaluation aimed to assess service users’ perspectives on the potential impact of the Hub on their interactions with their practitioners. The design of the evaluation was guided by a lived experience group and informed by a rapid evidence review. Data were collected from people with lived experience and practitioners through surveys, interviews, and focus groups, and an analysis of activity data was generated through the use of the Hub. The rapid evidence review on online harm; online interventions for suicide and self-harm; and usability, user engagement, and implementation of online mental health interventions informed the development of survey instruments and topic guides for interviews and focus groups. Common themes found in the evidence review were the pervasive and dual nature of online communications and content, the fluid sources of informal and formal mental health support, and the individual, technical, and social factors shaping the use of online interventions. The importance of engaging in discussions about online harm and involving people with lived experience in designing interventions was also apparent from the evidence review and taken forward into the instruments to gather data for the evaluation.

### Ethical Considerations

Focusing on the experiences of practitioners and people with lived experience regarding online harm, the study needed ethical approval from the researcher’s institution. This was granted after following the standard procedures of reporting the planned research and its methods to the school’s ethical board (School of Business, University of Leicester; approval 42217).

All participants of this study were provided with information about the evaluation and their voluntary participation, and were asked to consent to participate. This was obtained from all participants either by ticking respective boxes in the online survey or by filling in written informed consent forms. Participants who had not provided their consent before a focus group session or interview did so before the start of the focus group or interview. Their verbal responses were audio recorded, saved, and encrypted on the institution’s online storage service. An informed consent form was, in this case, filled in and signed by the researcher on behalf of the study participant. One online survey participant denied consent despite completing the survey. Their replies were deleted and excluded from analysis. As participation in the study and its components was voluntary, no compensation was paid to any of the study participants. Participants of focus groups and interviews were informed that all collected data would be anonymized and identifiable information deidentified before analysis and publication.

### Recruitment

Two surveys scrutinizing experiences and expectations of the Hub were circulated through Samaritans’ existing networks of practitioners and agencies supporting individuals at risk of harm or suicidal behavior, as well as through their lived experience networks that consisted of individuals with relevant knowledge through experience around the subject of online harm. The surveys also aimed at facilitating the recruitment of interviewees and focus group participants (all instruments used for this evaluation such as surveys and interviews or focus group topic guides are available in Multimedia Appendix 1). However, initially, very few practitioner participants (n=4) were recruited who could offer experience of engaging with the Hub. In response, the Samaritans, in collaboration with the evaluation team, offered three webinars in March 2024 as an alternative modality to promote engagement with the Hub and deliver the online training content while recruiting evaluation participants. The webinars had a high uptake; each webinar offered 500 slots, which were all prebooked and had a waiting list; at minimum, every session was attended by more than 50% of those who had signed up. A total of 920 people attended the three webinars at peak times. All participants were invited to complete a survey at the session’s end by displaying a QR code in the webinar’s presentation and sharing a survey link in the webinar’s chat. The survey also inquired if participants would consider attending a follow-up focus group, interview, or both.

### Data

Activity data from the Hub showing usage between November 2022 and December 2023 were collated through analysis of Google Analytics data charts to which access was granted by the charity. The lived experience survey was circulated through the Samaritan’s lived experience networks and received 45 responses, and 6 people with lived experience attended two focus groups (however, one focus group was unfortunately terminated prematurely due to technical issues and is thus not considered as data for this paper). The Samaritans and the evaluation team circulated an evaluation request for interviews through their practitioner networks, which resulted in 4 practitioner participants. The practitioner survey administered through Microsoft Forms at the end of each webinar session received 368 responses, and as a result, a further 5 practitioners were interviewed, and 7 practitioners attended three focus groups. Interview and focus group participant recruitment came to an end after this (Table 1). All surveys applied in this evaluation were analyzed for descriptive statistics analysis only using Microsoft Excel producing charts with frequency analysis of the respective answers to questions about usage of and expectations toward the Hub. However, as this paper aims to discuss the best-suited delivery modality for training on online harm, we mainly focus on presenting qualitative data here. The importance of the delivery modality was a result of the analysis and only emerged after noticing a significant uptake of the webinars, which were themselves a mitigation strategy for the low uptake of the Hub. The webinar survey was prepared before this became apparent and hence did not survey any relevant data in this regard that could be presented here. Both lived experience focus groups and practitioner focus groups were informed by results from the preliminary analysis of the respective survey data, for example, through scrutinizing some of the results further. The practitioners’ interviews, however, followed a topic guide that was already drafted before the webinar survey was applied and was instead informed by the results of the evidence review.

The interviews and focus group sessions for this evaluation followed semistructured interview topic guides (see supplementary appendices) and were conducted online using Microsoft Teams between December 2023 and April 2024. They were recorded, and verbatim transcripts of each were produced and thematically analyzed [36]. All qualitative data were analyzed by the first author, results were presented to and, if needed, challenged by the coauthors. As this process did not include analyzing the data originally by more than one researcher, no measurement for intercoder reliability was obtained. Transcripts were thematically analyzed using the qualitative data analysis software MAXQDA (version 11.2.5; Verbi GmbH); initial coding was undertaken to identify common experiences of using the Hub; this was then compared with the concept and components of CoPs, with the results presented in the Results section.

**Table 1.** Data sources and participants’ details.

Data source	Participant perspectives/roles	Number of participants/respondents	Data
Focus groups	People with lived experience of online harm resulting in either self-harm and/or suicidal ideation	5 (4 male, 1 female)	1 transcript of 55 min of audio
Focus groups	Practitioners from voluntary sector, NHS^a^, public health, local authorities, etc	7 (2 male, 5 female)	3 transcripts of combined 146 min of audio
Survey	Practitioners from voluntary sector, NHS, public health, local authorities, etc	368 (sex/gender was not collected)	Free text/Likert scale responses
Survey	People with lived experience of online harm resulting in either self-harm and/or suicidal ideation	45 (27 female, 11 male, 4 nonbinary, 3 preferred not to say)	Free text/Likert scale responses
Qualitative interviews	Practitioners from voluntary sector, NHS, public health, local authorities, etc	9 (3 male, 6 female)	9 transcripts of combined 385 min of audio
Hub activity data	All users	—^b^	Quantitative internet usage data

a NHS: National Health Service.

b Not applicable.

## Results

### Overview

**Figure 1.**
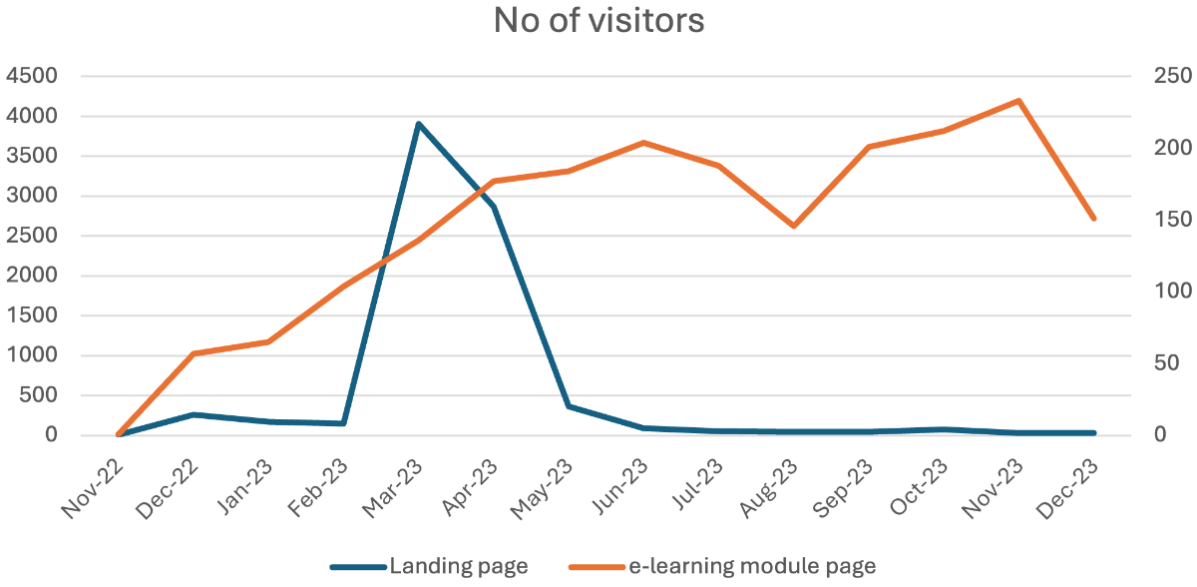
Number of online visitors to the Hub’s landing page and e-learning module between November 2022 and December 2023. Samaritans Online Safety Hub data from Google Analytics.

Although the analysis of the quantitative and qualitative data collected through surveys, interviews, and focus groups revealed that the Hub resources were deemed very useful by those who had used them, analysis of the activity data indicated there had been relatively limited uptake of the Hub. The launch of the Hub in late 2022 resulted in some uptake of online training (with a total of 539 module launches between November 2022 and April 2023) and up to 3900 visits to the Hub web pages each month between March and May 2023 (visits to the guidelines landing page). However, after this peak in user visits, the number of users declined and remained on a low monthly average (between 20 and 90 visits from June to December 2023; Figure 1).

Practitioners represented a wide range of organizational settings and roles, including frontline mental health workers, people with organizational safeguarding roles, student counseling services, and freelance online consultants (Table 2).

**Table 2.** Organizations of the online safety webinar participants (N=368).

Category	Value, n (%)
Charity, community, or voluntary sector	98 (26.6)
Education provider (including school, college, or university)	80 (21.7)
Local authority	52 (14.1)
NHS^a^ Trust (acute, community, or mental health)	98 (26.6)
Other	22 (6.0)
Primary care	17 (4.6)
Missing answer	1 (0.3)

a NHS: National Health Service.

The comparatively high demand for the webinars offered by the Samaritans, which used the same resources as were provided on the Hub, indicated that the modality by which practitioners might engage with those resources was of great importance. We found that synchronous or “live” webinars were experienced as a more sociable form of learning than individual self-directed learning, which was what the Hub had previously provided. We applied the concept of CoPs to the qualitative evaluation data to consider the importance of different modalities of learning for practitioners’ experiences and use of the Hub. We present our findings in the sections about the sociable nature of learning, community membership, learning about the sensitive topic of online harm, and incorporating learning into practice.

### Sociable Learning

The comparative popularity of the webinars compared with the use of the website and e-learning resources indicated a preference for the more sociable experience offered by participating in a webinar to that available from independent learning. Learning from and engaging with peers was found to be important to participants in the webinars:

*I also enjoyed the Q&A, the interaction, and the interactive aspect...Because that opens up kind of again that less presentation style, kind of dialogue about something, it makes you back up a bit when you're in an online webinar because you want to see what’s coming in from other people sitting behind their desks. […] I also think it gives a perspective of what other people think of in the room, aside from the facilitators.*

[Practitioner 7, public health officer for community engagement within local authority]

Listening to the experiences of other members of this new community was felt to be a valuable component of the webinars:

*‘I just feel like I do normally enjoy that part of benefiting from hearing other people’s pressing questions or examples because it then makes you think even more widely outside the box as to what it means.*

[Practitioner 7, public health officer for community engagement within local authority]

When asked how the Hub might develop and improve, practitioners suggested more opportunities to facilitate the sharing of issues and challenges on the subject, for example, through discussion forums. Practitioners identified social learning as important in allowing them to incorporate new knowledge into their practice; when asked what they might do differently as a result of attending the webinars, some suggested sharing the resources with colleagues, for example, in team meetings. The social experience of participating in focus groups and interviews was also noted as useful in learning and reflecting more on the issue of online harm.

Some participants noted the importance of creating a comfortable learning environment, aware that the course content could trigger negative emotions. This sense of creating a caring and protected community was noticed explicitly by one practitioner who summed up her webinar experience:

*I always really enjoy the way the courses [sic] are delivered. It’s obviously such a really sensitive way. They have a real focus on not only learning what our patients are experiencing but actually, I really like how there’s always a focus on actually looking after ourselves as well. Which is just a really nice touch, I think. And obviously, there’s such a wealth of experience and knowledge there that it just always feels like a really warm environment where you can ask any sort of question, and it’s not going to be like a silly question or anything. So I thought the information was really interesting. I definitely learned a lot [...] And again, there was that sense of kind of no question was off limits.*

[Practitioner 8, health and well-being coach in NHS GP partnership]

The ways in which participants indicated they valued the webinars align closely with the benefits associated with CoPs. This social aspect of learning during webinars was also experienced in focus groups, where participants reflected on the benefits of having time to discuss online harm with other practitioners.

### Community Membership: Who is a “Practitioner”?

A wide range of people are potentially involved in counseling people about online harm in relation to self-harm and suicide. The pervasiveness of internet access, and therefore the potential for online harm, was a feature of people’s personal as well as their professional lives. The term “practitioners” was used to encompass the diverse people who offer support around the issues of self-harm and suicide, and the online resources were titled “Guidelines for Practitioners.” This generic term avoided specific job roles to deliberately encompass people working in a range of organizational settings, including NHS services, local authorities, and the voluntary sector. Despite the use of this general term, some people were uncertain if the Hub (and in particular the e-learning module) was intended for them, as they did not recognize themselves as practitioners because they did not work in mental health services or NHS organizations. Although the content was designed for and valued by a broader range of audiences than mental health practitioners in the NHS, the process of accessing the e-learning module involved creating an account on the NHS online platform, which appeared to require an organizational background in health-related professions. Similarly, while many people signed up for the webinar, some were initially doubtful if it was aimed at them, as one interviewee said:

*There’s something about the comms about who is a practitioner and who this learning is for. So, I'd say that needs to be a bit clearer. I wasn't sure when I joined the webinar. Am I OK to be here? Basically, because I'm a consultant supporting people who are helping people at risk of self-harm and suicide. So, hopefully, it was OK for me to be there, but it did feel a little bit like I was surrounded by a bunch of people who had very different jobs to me, and I just didn't know whether I was in the right place. So, there’s something about the comms around that.*

[Practitioner 5, freelance online community management consultant]

Recommendations from practitioners aimed at improving the Hub included greater clarity about the target audience. One way to achieve this, as suggested by participants, was to create more tailored and adapted versions of the resources. For example, different settings (cultural backgrounds and age-related stages) in which people may encounter harmful online experiences could be specified in the Hub, which could both reflect the specific needs of service users and the community of practitioners. A more personalized approach would align well with other practices:

*I think personalisation is a huge place of where we're at. Our well-being network operates on the same basis. People are given choices of topics, age groups of people they work with, and the areas they work in. And so the only information they receive is related to it directly to those. So there’s a lot of information they miss out on that other people get based on their individual choices. So questions like why are you here? You know what do you do? Do you support people? OK, are you aware? Click on these areas that you're already aware of, and then it narrows everything down and deletes what’s not, you know, necessary.*

[Practitioner 4, health improvement specialist in mental health team of local authority]

Participants contemplating how to raise the issue of online harm in their professional practice made connections with how they might talk about it with other people in their lives, particularly younger people in their roles as parents, aunts, or uncles. As one participant noted, their learning from the Hub was of broader social benefit:

*As a person with regular contact with young people in other roles and capacities as a parent, as an auntie, as a [youth group] leader, as you know, other things that I do in my role, in my life, I would say it probably has, like I might not have previously thought, to ask somebody about what they’re accessing online and how that’s making them feel and I feel confident to start that conversation with someone. So I would say yes, it has made me feel a little bit more confident and highlighted that that’s a conversation I might need to have.*

[Practitioner 2, public health practitioner and suicide prevention lead]

Through their engagement with the Hub, practitioners recognized the importance of raising online harm with people, whether those seeking support from them as practitioners or other people in their lives. In this respect, the Hub had achieved one of its key aims. However, the low uptake of the Hub appeared to be related to people not being sure if they were the intended audience. This has implications for how a CoP is created and how resources need to be targeted to those who might be able to use them.

### The Sensitive Topic of Online Harm

The reason for people engaging with the Hub—online harm—was commonly reported to be a sensitive and challenging topic for people to talk about. Similarly, the topics of self-harm and suicide are generally considered to be difficult and sensitive. Expertise in dealing with sensitive issues generally, familiarity with online social life, and a shared understanding of the rationale for raising online harm all contributed toward helping practitioners feel more confident in having discussions about online harm.

We found that some practitioners were not used to raising the subject of online harm, which they connected to their own lack of familiarity with the different platforms used and less personal use of, for example, social media. Some of these practitioners associated their lack of familiarity with online life with being from an older age group. However, when they were already skilled and experienced at talking about the sensitive topics of self-harm and suicide, they were able to reflect on their existing competence in discussing difficult issues when considering how to raise the additional topic of online harm. This gave them some confidence when planning to incorporate the topic of online harm into their practice, as one practitioner described:

*I ask all the generic, you know, do you have any thoughts to harm yourself and the scale and what’s the intent and how often and frequent, but I don't actually ask a question about accessing material online and have they done so...I’m sort of thinking...what sort of question would be relevant to maybe add to that? To capture more people that maybe are using the internet or being exposed to content that is maybe not helpful. I am very familiar with the Samaritans because I've done their training previously as a Samaritan. I feel really confident to ask people about suicide*....

[FG1 participant 3, private counselor and supervisor]

One practitioner (who reported they were very familiar with a wide range of social media platforms) explained that they would routinely include discussion of online activities or triggers when talking to their clients:

*...it’s something that we do so routinely that they start to feel comfortable with us doing it, that they just sort of like, it’s kind of like asking if, hey, are you OK? Is there anything you want to talk about that we asked him? Hey, the content that you've consumed on the internet, do you want to talk about it and how does it make you feel? And they do just answer it like any other question that we ask of them.*

[FG1 participant 2, mental health worker in high-dependency rehab unit]

A central message of the Hub was that talking about online harm can be helpful and does not have to be avoided. This echoes much of the guidance about talking to people about suicide and self-harm; talking openly about these topics can help people explore their feelings and avoid secrecy and judgment [35,37,38]. The inclusion of videos of people talking about their lived experience of self-harm and online harm in the Hub was helpful in this respect, as one participant reported:

*[T]here was that balance of some online access being supportive to somebody...who might self-harm and they might want to talk to somebody else, you know, to kind of let that out. To talk, you know, as an outlet, if that makes sense. But also there was that other extreme of, you know, I have seen stuff in media where by somebody’s encouraging somebody to, you know, cause extreme harm to themselves or indeed end, you know, end their life so and so yeah it was I found it informative and educational.*

[FG3 participant1, operations director for statutory advocacy services, organizational safeguarding lead]

Some participants suggested that resources, such as information and coping mechanisms, should be developed and provided directly to people at risk of online harm in relation to suicide and self-harm, in addition to the resources developed for practitioners. Practitioners who suggested this were aware of their limitations in reaching all the people who might be affected by online harm and felt that the ubiquitous nature of online life and potential harm needed further action to address. One participant framed this in terms of a “warning” that should be included when people buy a mobile phone, for example, alerting them to the potential negative aspects of accessing social media. The pervasive nature of online harm and the breadth of the population who might be affected were acknowledged by many participants. Sharing experiences during focus groups was also valued by participants, particularly by those who learned that others were routinely raising the topic of online harm in their work.

### Incorporating New Knowledge Into (Shared) Practice

A raised awareness about the seriousness of online harm was frequently described as a result or outcome of taking the training and engaging with the Hub, for example, when asked about the main benefit of the Hub, one participant responded:

*...bringing it up to people’s attention because I am realising more and more that the internet has a big impact on our lives, and that can be positive; it can be negative. But I think just mainly putting it out there so that people are aware to consider it and not skim over it or think about the impacts because really people are using it in different ways. Some people might not even use it at all, but it’s really hard to avoid the internet now.*

[Practitioner 3, public health practitioner & previous frontline mental health practitioner]

Raised awareness was reported as a first step to eliciting change in the practitioner’s practice. In both interviews and focus group sessions, participants shared how they had changed their practice or planned to change their practice as a result of engaging with the Hub. It was evident that some practitioners not only reflected on the impact the gained knowledge would have on their practice but also on how they could share and recommend what they learned to fellow practitioners in their organization and beyond. For example, when asked if the training had already improved the experience of interacting or engaging with service users, one practitioner answered that their service would now regularly inquire about adverse online experiences with all their service users in the future:

*...now we are thinking of making it a part of our initial assessment. So there is an initial assessment that we do for us to measure what kind of crisis and what level, and taking this component and putting it there already, I think it'll be quite helpful for us because it would also give us a broader overview of where that person is and what kind of content they're engaging.*

[Practitioner 9, manager of crisis hub]

Both the survey and focus group session with people with lived experience indicated the important role of being asked about online behavior by their practitioners. During focus groups, practitioner participants started to formulate specific conversation starters and questions as they reflected on how their engagement with the Hub could impact their practice, for example:

*…the question which I think would be hugely beneficial even to friends, family or clients is to ask about is there anything you know that distresses you about the use of the internet and that can come up, that can be brought in at any time, more as part of the intake.*

[FG1 participant 3, private counselor and supervisor]

Shared learning was expressed during the focus groups, as other participants agreed they would use similar questions and discussed how they found the evaluation itself useful in articulating their learning and changes to their approach.

The evaluation of the Hub showed that the key messages of encouraging and supporting practitioners to raise the topic of online harm with people they were supporting with the issues of self-harm and suicide were well received and likely to lead to changes in practice. Practitioners articulated a number of ways in which they planned to incorporate their learning from the Hub into their work. Despite working in diverse organizational settings, participants identified a common purpose and a shared interest in learning together about how to address online harm for people at risk of self-harm and suicide. The emergent CoP that engaged with the Hub was made up of people with shared concerns and experiences about addressing sensitive and difficult topics. Whereas the provision of online resources alone was insufficient in generating wide uptake, a more social approach to learning, which we conceptualize as a CoP, engaged practitioners more effectively.

## Discussion

### Principal Findings

This evaluation of the Samaritans’ Hub, derived from activity data, survey data, qualitative interviews, and focus group sessions with practitioners, shows that the resources of the Hub were found to be helpful in informing and creating awareness about online harm by relevant practitioners. However, uptake of individual e-learning and downloadable resources was low in comparison with the opportunity to learn about the resources in the more sociable modality of synchronous or “live” webinars. We interpret these findings as an indication of the value and benefits of participating in a CoP. Our work adds to the previous literature on CoPs [11-17], to the evidence on interventions aimed at addressing online harm in relation to self-harm and suicide [19,22,31,35], and to the broader knowledge about how to implement online interventions [5,6,9,39].

Previous research identified the importance of being explicit about whom a CoP may benefit and fostering a sense of belonging among its members in order to prevent early dropout [40]. This was particularly important for the online CoP that emerged as people in diverse roles engaged with the Hub via the webinars. Uncertainty about membership of the community was linked to being unclear about the target audience of the intervention. It is important to *“*get the pitch right,” as one interviewee noted (participant 2, public health practitioner and suicide prevention lead). Uncertainty about membership was resolved by participating in online webinars where people could share experiences and engage in a process of collective learning. While the CoP evolved from a shared interest (in this case for online harm, suicide, and self-harm), ways of identifying members, including the language used (eg, “practitioners”), and processes of sign-up (such as to e-learning from an institutional account) could impede community forming. Previous research has analyzed multiple ways in which online CoPs form [39], sometimes as a planned and organized endeavor, and at times, they are set up to stay in touch with other professionals, for example, after meeting at a conference [39]. An important feature of this CoP was the need to deal with a sensitive topic, supporting people with online harm, self-harm, and suicide, and the safe space that was provided for practitioners to discuss unfamiliar or unknown areas of the online world. In this context, the care taken by the webinar facilitators to acknowledge the potentially distressing nature of the subject matters of online harm, self-harm, and suicide, and the need for everyone involved to protect their own well-being was noted as an important strength by participants. This aligns with previous research indicating that delivering sensitive topics through sociable learning environments offers several benefits, which may explain the preferential uptake but may also have some drawbacks [41]. It fosters social interaction among participants, leading to social presence and the emergence of a sound social space, which in turn explains the quality of the learning experience. However, while it is essential for students in health and social sciences to receive education on sensitive topics such as sexual violence [42] or, in our case, to educate practitioners about online harm, increasing awareness of these topics can elicit emotional or distressed responses among learners [43]. Learning together through a CoP allows for peer and facilitator support.

Positive feedback from people who had engaged with the Hub about the contents and key messages indicated that this was an intervention that could help address online harm related to self-harm and suicide. The integration of knowledge about online harm with preexisting expertise in supporting people with self-harm and suicidal ideation was facilitated by the sociable learning of webinars and the sharing of knowledge about online life, such as social media, endorsed the Hub resources.

The online intervention shaped and informed the development of a CoP. However, the CoP also shaped the intervention. Changing the delivery modality of the intervention during the evaluation process to recruit more evaluation participants caused an increased uptake. What started as a mitigation strategy for low uptake of an evaluation resulted in a permanent addition to the online resource, with more webinars on the subject now being held regularly by the charity, resulting in a total number of about 1800 participants educated on the subject through the webinar by the charity in 2024. Furthermore, the question and answer sections of these webinars were especially emphasized as suitable ways to discuss and receive suggestions for individual experiences on the subject and to raise awareness about the issue. When a CoP simultaneously interacts with facilitators responsible for the webinar content, this can create a short feedback loop for the participants. The area of online harm is a quickly evolving field, necessitating such rapid feedback loops to keep the intervention, in this case, the Hub, responsive and up to date, which could facilitate knowledge uptake into practice.

### Limitations and Strengths

Initially, we received very few responses to the survey, limiting our ability to evaluate the Hub. A strength of the evaluation was the flexibility of the charity in developing a new method of engaging people in the Hub and the evaluation through the delivery of webinars. This resulted in more responses. However, those responses were from webinar participants rather than practitioners who took the online training immediately after it became available. As such, it was not possible to evaluate how effective the Hub had been in changing practice, and further follow-up would be required to achieve this. An additional strength of the evaluation was the ongoing support and engagement of people with lived experience in the evaluation process who provided insights into what was helpful and important for those experiencing online harm, self-harm, and suicidal ideation.

### Conclusion

Online interventions that seek to facilitate training and implementation of evidence-based prevention strategies need to be supported by and are activated through collective learning processes such as CoPs. CoPs provide an infrastructure to support social learning and knowledge sharing, particularly in complex or uncertain contexts. Supportive, collective learning is particularly important when dealing with sensitive topics and concerns that are pervasive, such as online harm, and that are novel, for example, to people with less experience with internet safety and harmful online activity.

## Supplementary material

10.2196/72130 Multimedia Appendix 1 The lived experience survey, postwebinar practitioners survey, interview topic guide, and topic guide for focus group.

## Abbreviations

CoP: community of practice
NHS: National Health Service
