# Supplementary material for: Contribution of an Online Intervention to Developing Communities of Practice: Mixed Methods Evaluation of an Online Safety Hub to Address Harmful Online Content in Relation to Self-Harm and Suicide
Source: JMIR Form Res. 2026 Jan 14;10:e72130. doi: 10.2196/72130 (PMC12803438; doi:10.2196/72130)
Supplement: Multimedia Appendix 1 [file formative-v10-e72130-s001.docx]

## The Lived Experience Survey

(Q1) How often do you use the internet to engage with topics around suicide and self-harm?

(Q2) What is your primary source of information for mental health topics on the internet?

(Q2 other) If other, please specify:

(Q3) Do you feel safe when using the internet?

(Q3 Please specify) If it depends, please specify:

(Rank Slider Q 4) Would you agree to the following statement? “When I use the internet, I take care to guard my privacy.” Please rate on a scale of 1 to 5, where 1 = strongly disagree and 5 = strongly agree Please click on and move the slider to where you would rate yourself.

(Rank Slider Q 5) Would you agree to the following statement? “I am aware of the risks of internet use around suicide and self-harm.” Please rate on a scale of 1 to 5, where 1 = strongly disagree and 5 = strongly agree Please click on and move the slider to where you would rate yourself.

(Q6) Have you seen online material around suicide and self-harm that was distressing or harmful to you?

(Q6 please specify) If yes, please specify briefly what kind of content you have seen and under what circumstances you have seen it

(Rank Slider Q 7) How likely would you rate your reaction to the following scenario? “If I experienced something distressing or harmful online, I would talk about it with a mental health practitioner.” Please rate on a scale of 1 to 5, where 1 = extremely unlikely and 5 = extremely likely. Please click on and move the slider to where you would rate yourself.

(Rank Slider Q 8) Would you agree to the following statement? “I feel skilled enough to use the internet safely.” Please rate on a scale of 1 to 5, where 1 =strongly disagree and 5 = strongly agree Please click on and move the slider to where you would rate yourself.

(Rank Slider Q 9) Would you agree to the following statement? “In case I worry about things I see or do online, I know where I can get help and support to deal with my experience?” Please rate on a scale of 1 to 5, where 1 = strongly disagree and 5 = strongly agree Please click on and move the slider to where you would rate yourself.

(Q10) If you agreed or strongly agreed with this statement, can you briefly indicate where you would get your support from?

(Rank Slider Q11) Would you agree to the following statement? “Knowing that my practitioner can receive online training about internet use, suicide and self-harm would make me more likely to talk to them about my internet use around suicide and self-harm.” Please rate on a scale of 1 to 5, where 1 = strongly disagree and 5 = strongly agreePlease click on and move the slider to where you would rate yourself.

(Rank Slider Q11a) Would you agree to the following statement? “Knowing that my practitioner can receive online training about internet use, suicide and self-harm makes me feel safer.” Please rate on a scale of 1 to 5, where 1 = strongly disagree and 5 = strongly agreePlease click on and move the slider to where you would rate yourself.

(Rank Slider Q12) Would you agree to the following statement? “Knowing that my practitioner can receive online training around internet use, suicide and self-harm would improve my relationship with my practitioner." Please rate on a scale of 1 to 5, where 1 = strongly disagree and 5 = strongly agree Please click on and move the slider to where you would rate yourself.

(Rank Slider Q13) Would you agree to the following statement? “I feel supported by my mental health practitioner when I share my experiences of using the internet to explore self-harm and suicide."Please rate on a scale of 1 to 5, where 1 = strongly disagree and 5 = strongly agreePlease click on and move the slider to where you would rate yourself.

(Q14) What support do you expect from a mental health practitioner regarding internet use around suicide and self-harm?

(Q15) Would you consider taking part in a an interview or focus group (either online or telephone) to talk about the topic in more depth with one of our researchers? (please select all that apply)

(Q16) If yes, please provide a contact email address for a researcher to contact you with more information.

(LE Further Comments) Do you have any further comments you would like to share with us?

(PQ1) Do you have lived or living experience of any of the following?

Lived experience of suicidal thoughts

Lived experience of suicide attempts

Lived experience of self-harm

Have been bereaved by suicide

Experience of Samaritans service use

(PQ2) That’s great to hear! We would now like to ask you a few questions so that we can get to know you better. Which age bracket do you fall into?

(PQ12) Which of the following best describes your gender?

(PQ2) Which of the following best describes your sexual orientation?

If you prefer to use your own term, please provide it here:

(PQ3) Do you identify as trans?

(PQ5) Ethnicity:

(PQ5_4) If other ethnic group, please specify:

(PQ5_Prefernottosay) If you would prefer to use your own definition, please specify:

(PQ6) Which of the following regions do you live in?

## Post-Webinar Practitioners Survey


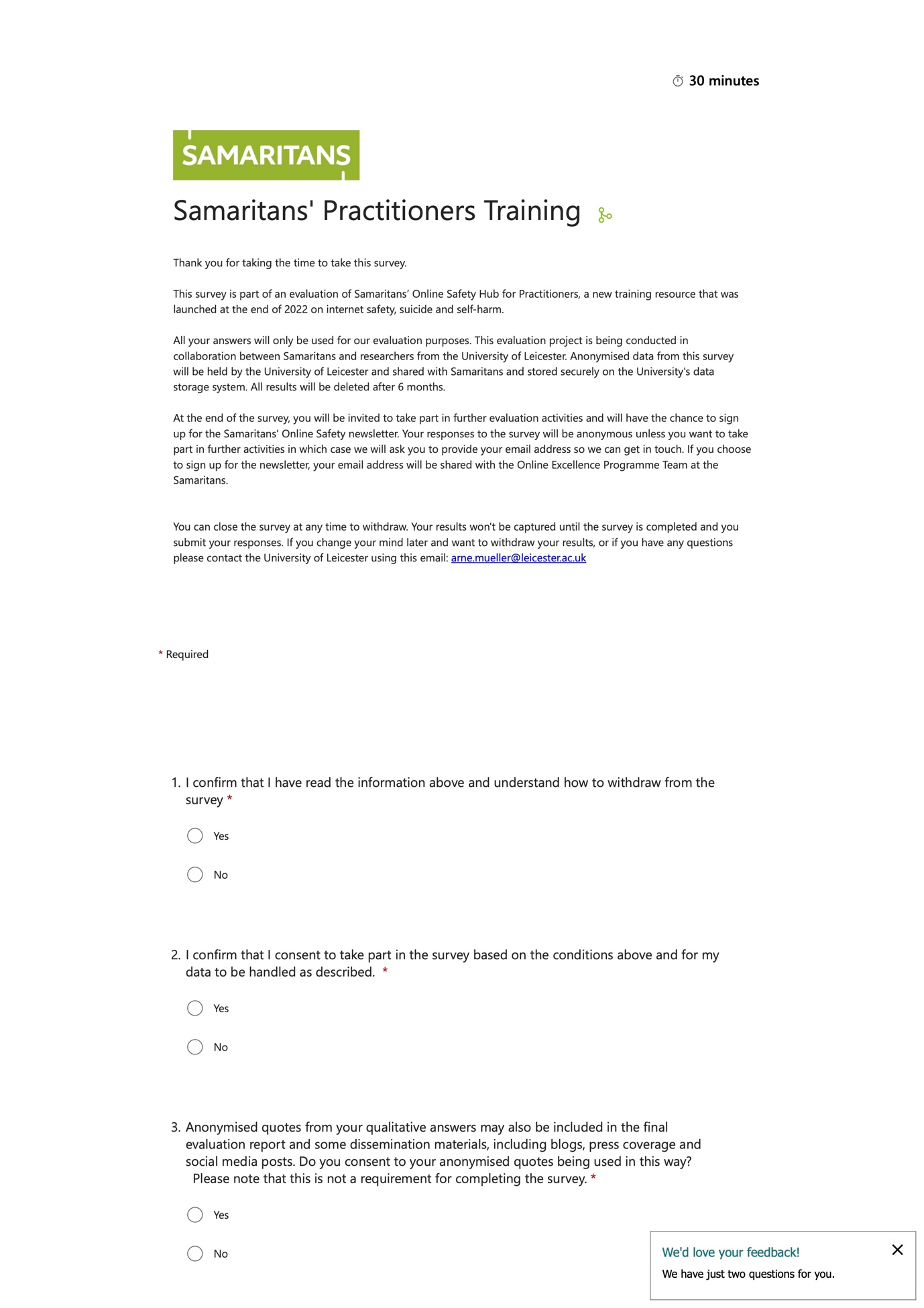


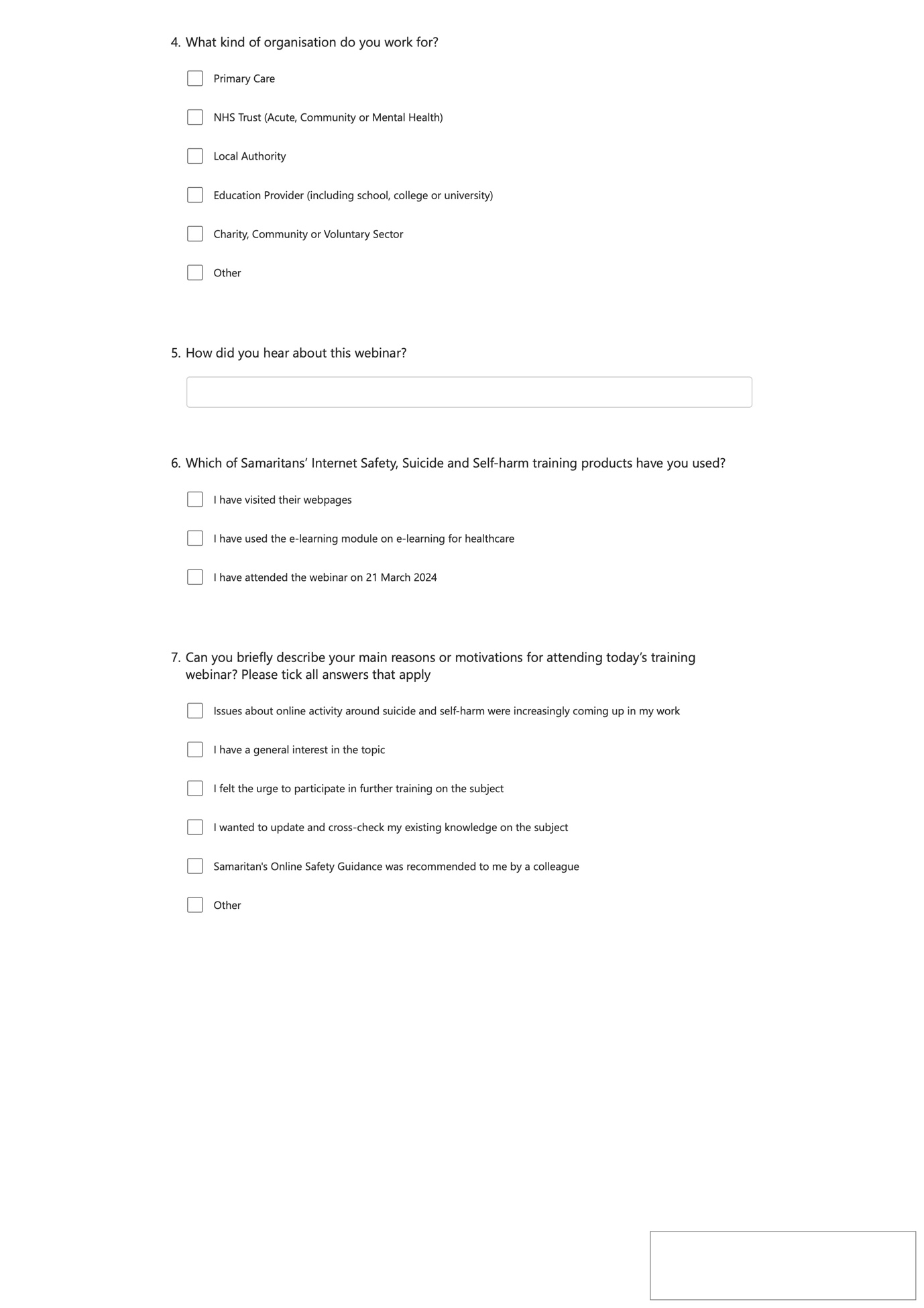


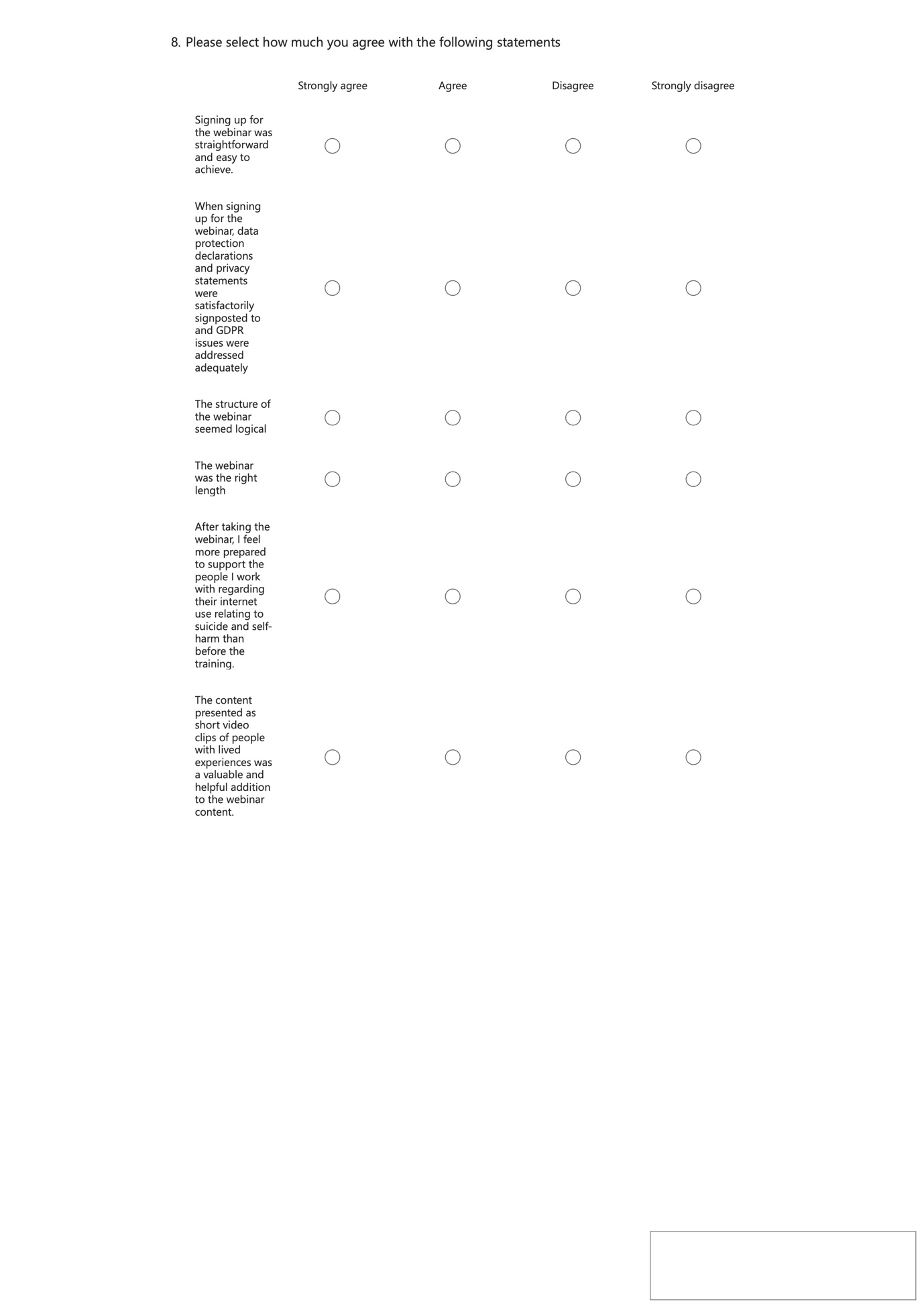


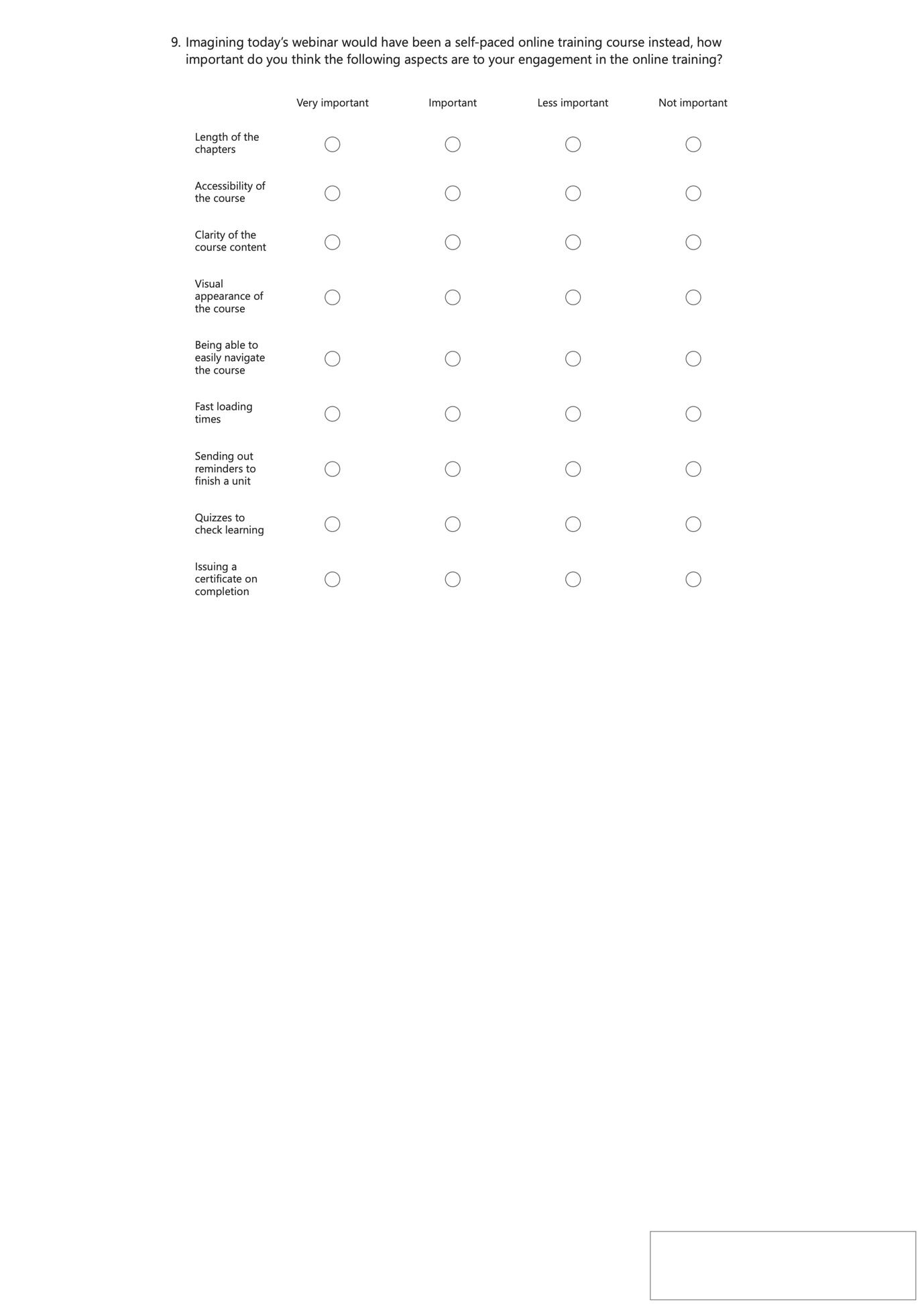


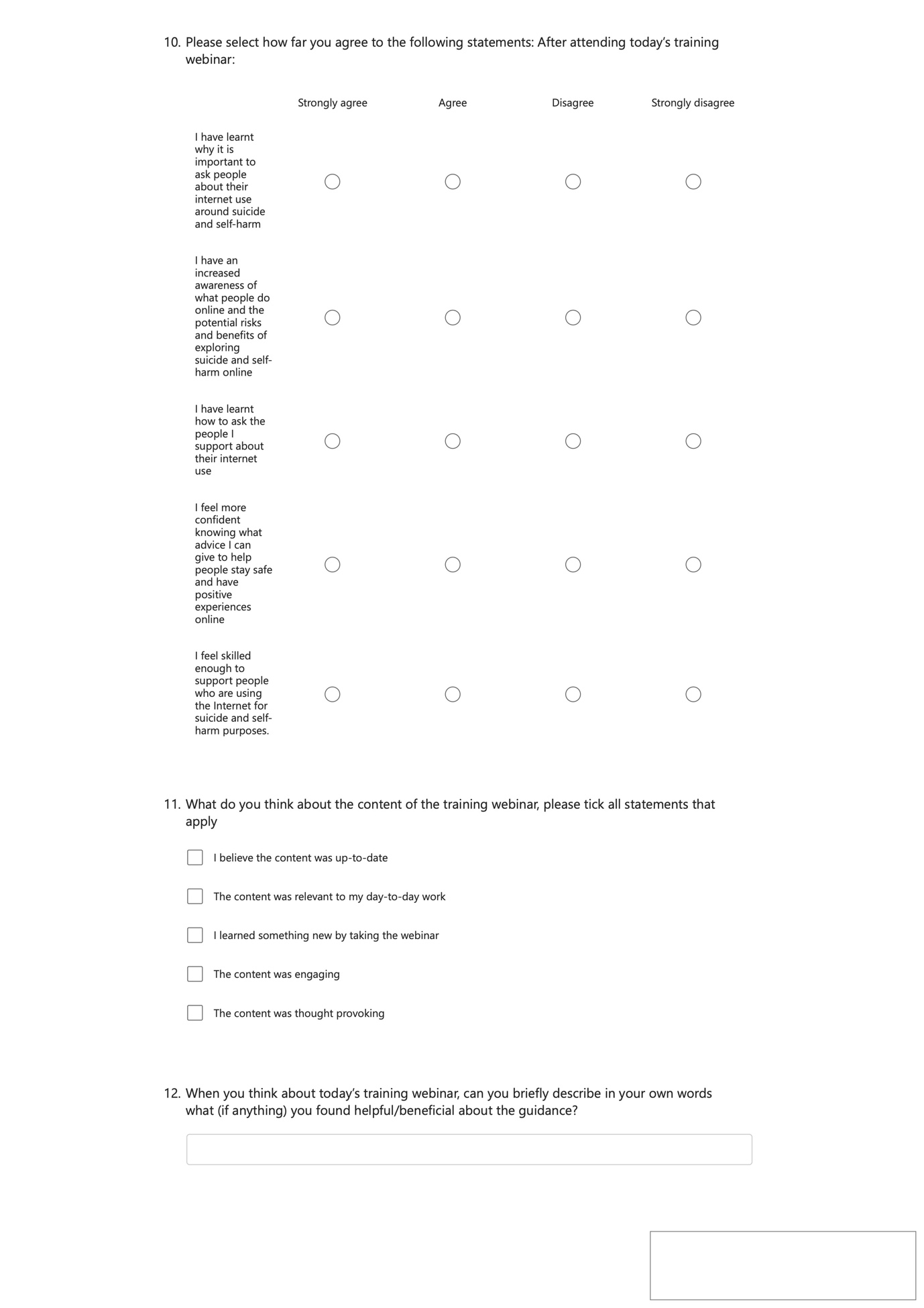


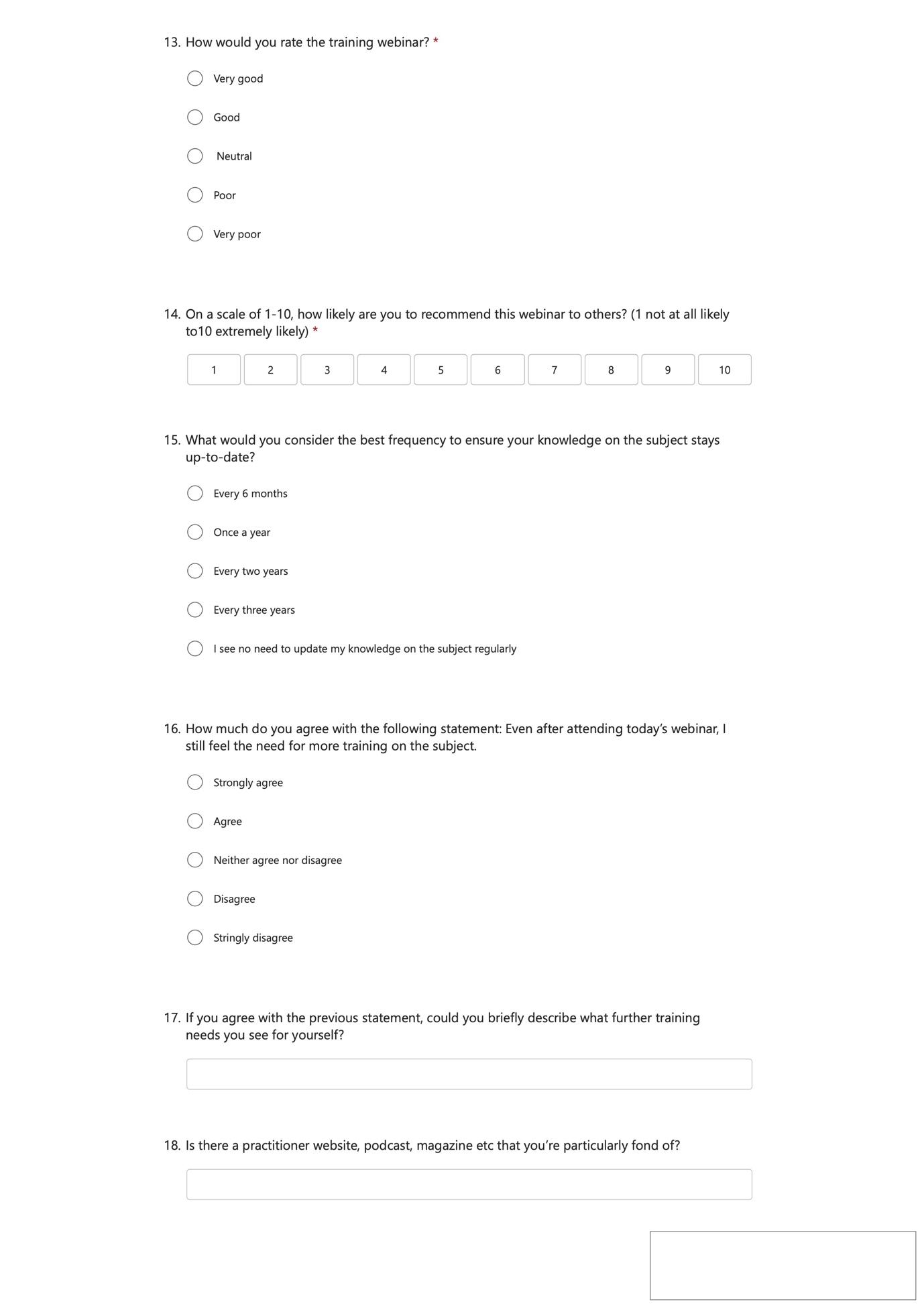


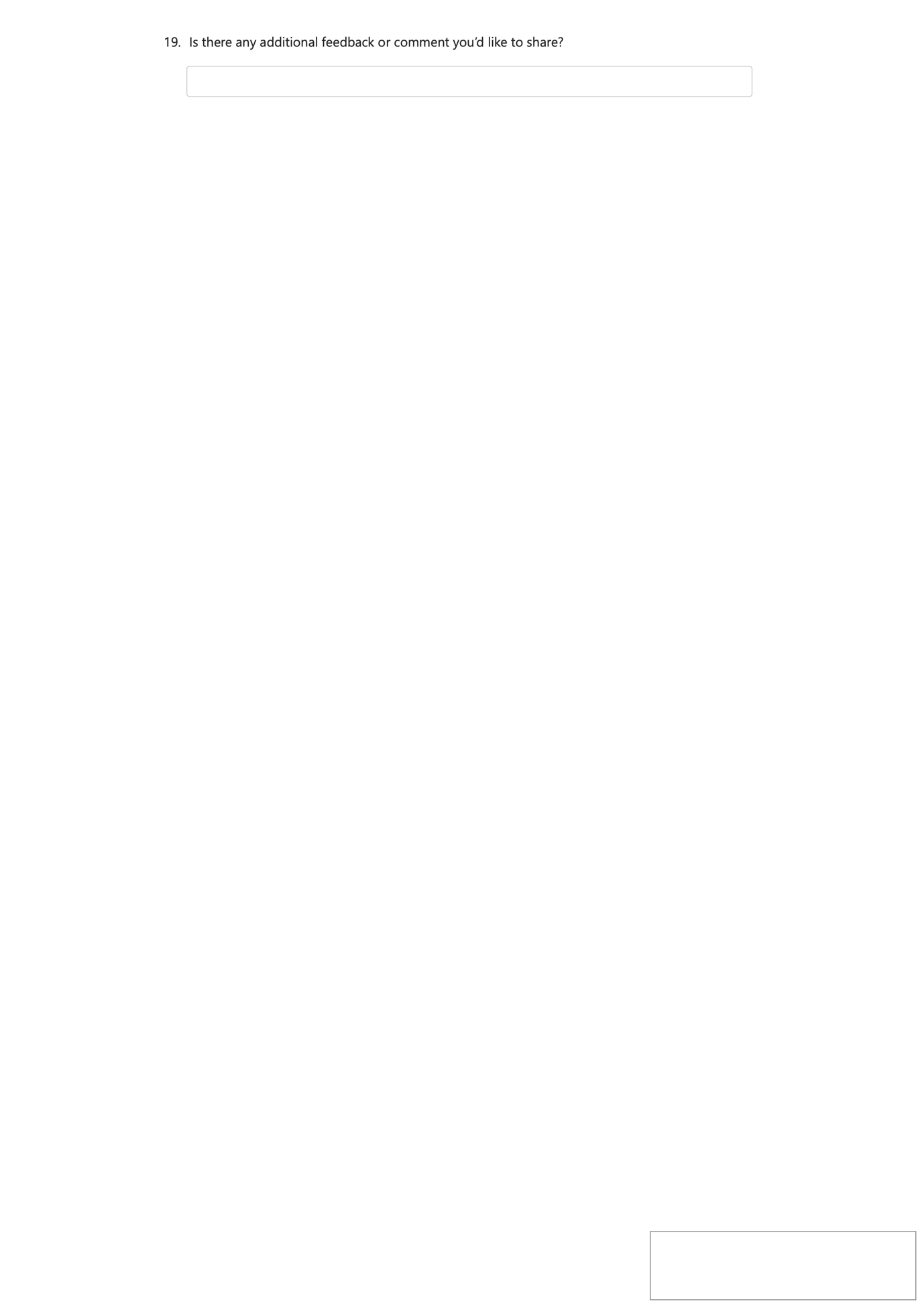


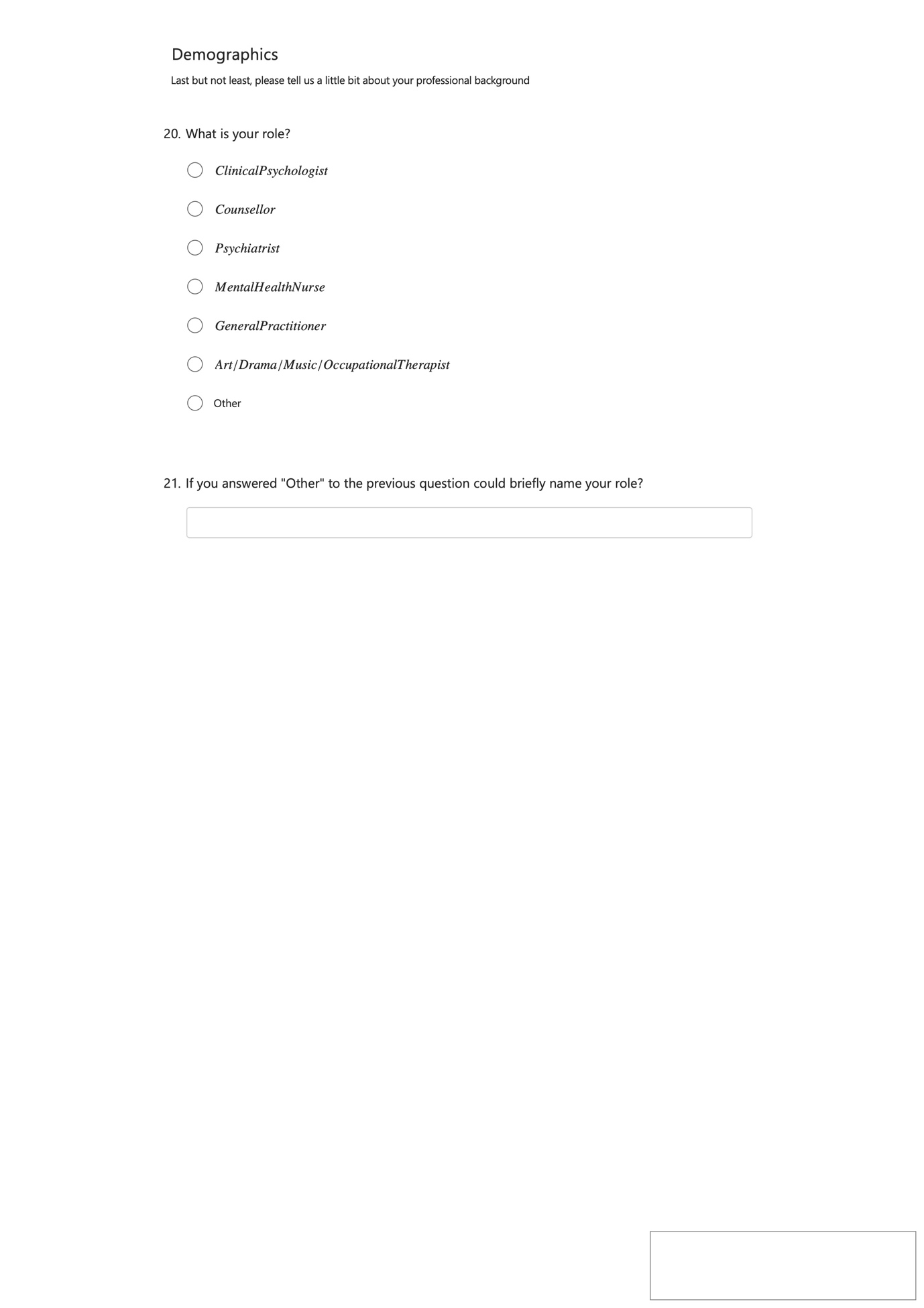


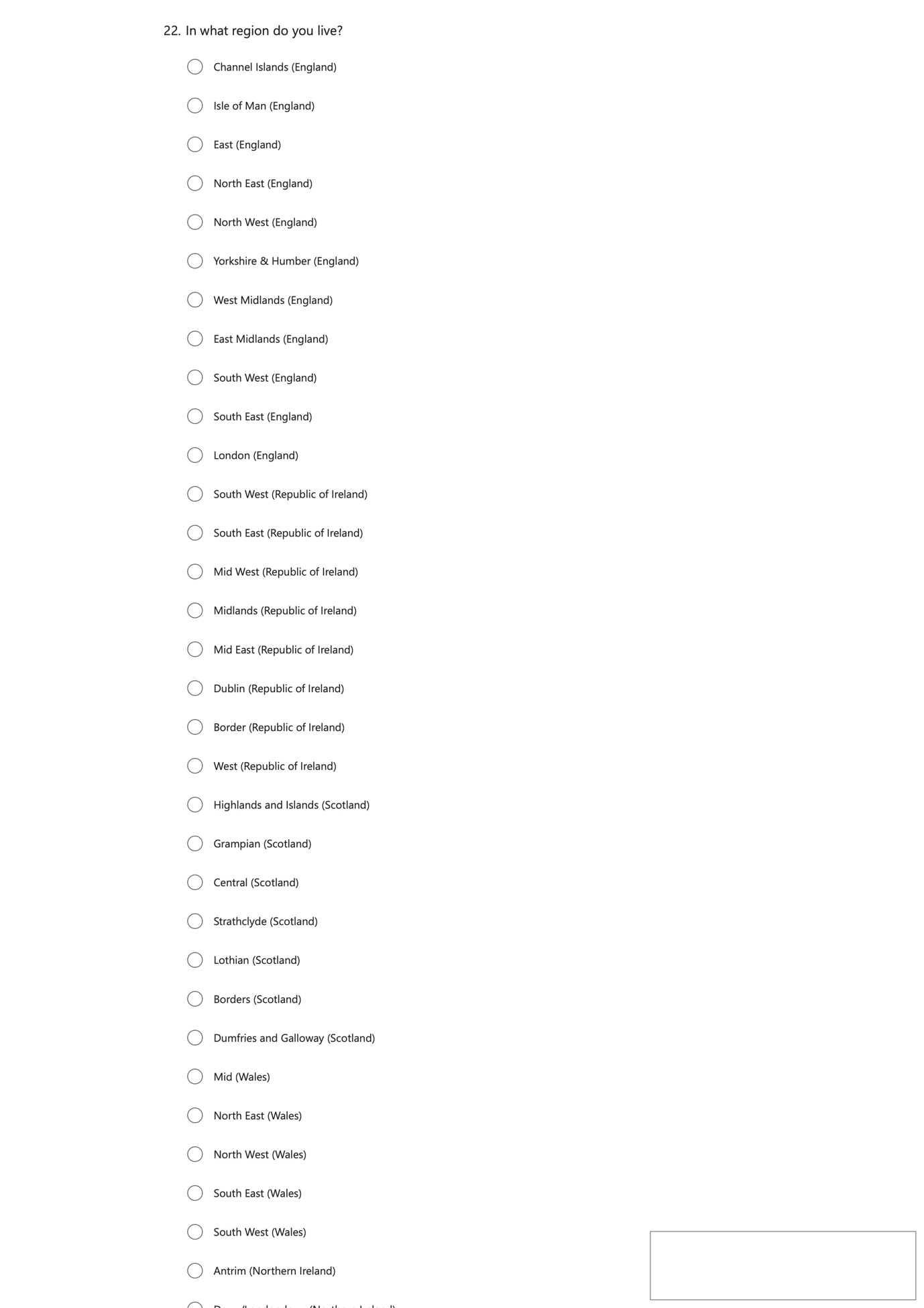


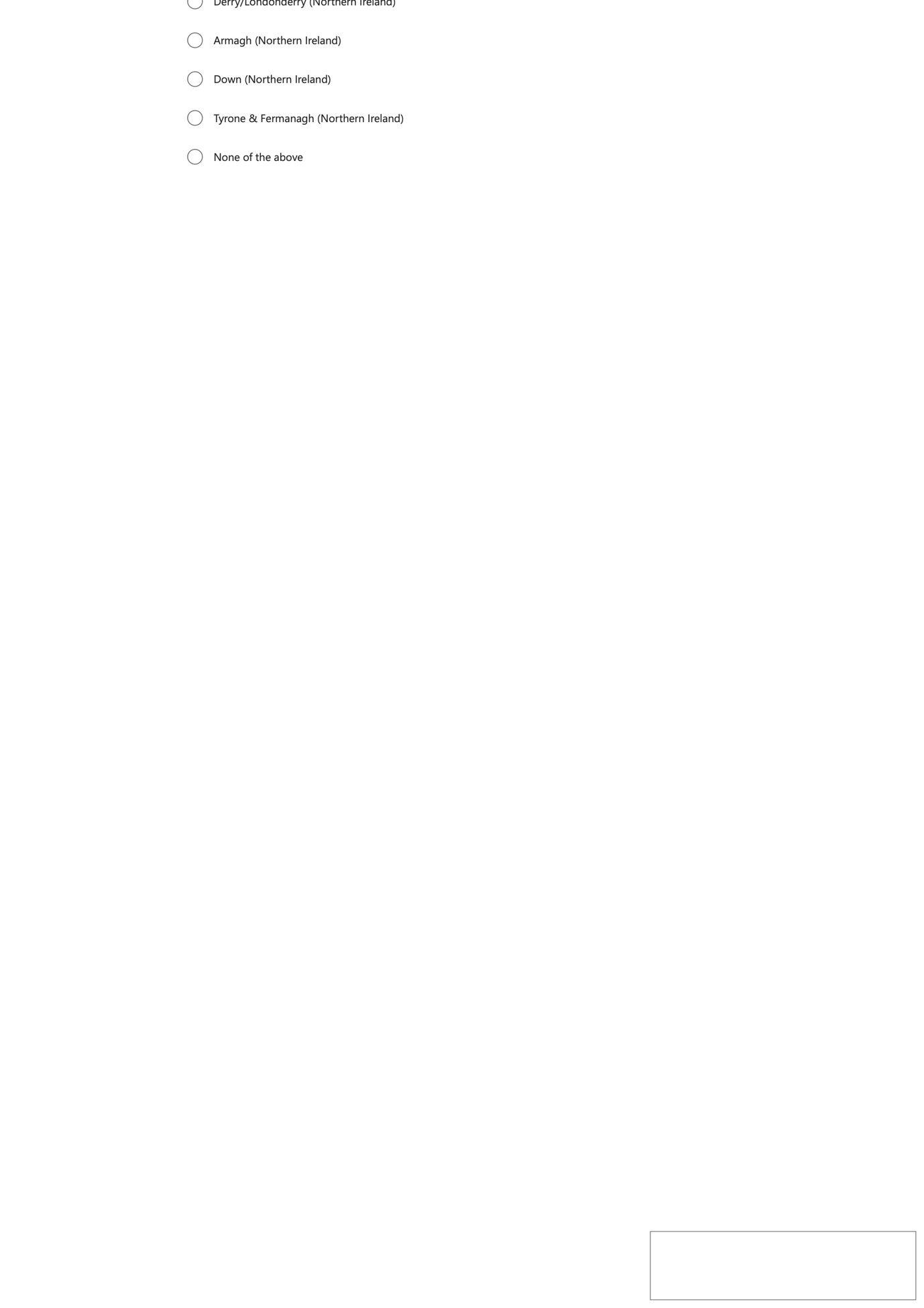


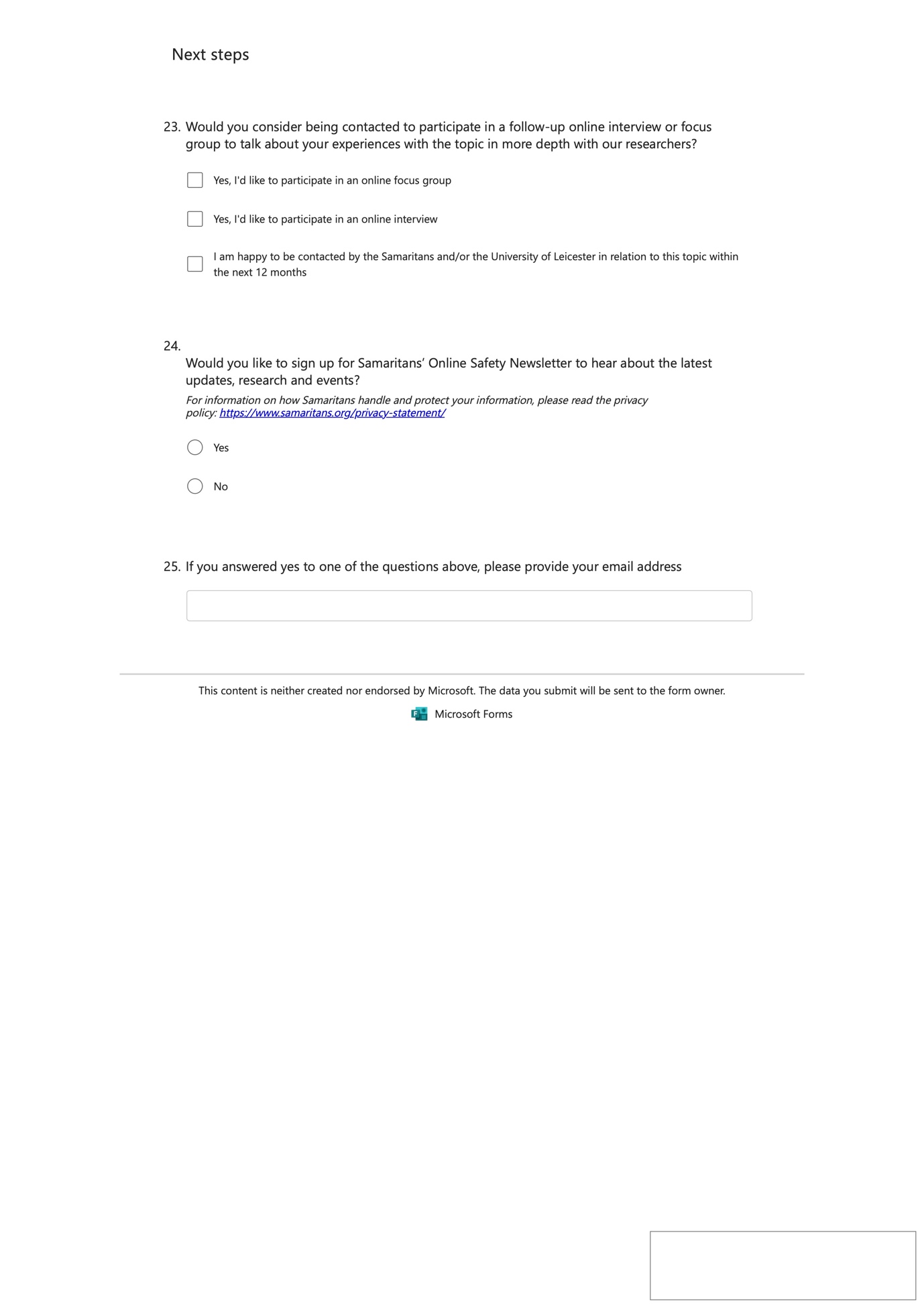


## The Interview Topic Guide

**Online Safety Hub Evaluation**

**Interview Topic Guide for Practitioners**

**Welcome and introduction**

Thank you very much for agreeing to participate in this interview. We have prepared a few questions that will help us to evaluate the implementation of the Online Safety Hub provided by the Samaritans. The perspectives you will share will have a direct impact on future versions of the hub, so we appreciate it if you contribute whatever comes to your mind regarding our questions. It is possible that some of the things we talk about might be difficult to discuss or upsetting, or that you simply prefer not to talk about. If we come across anything that might upset you or you do not want to talk about, just let us know and we are more than happy to skip the question.

**Seek consent to continue and to audio-record the interview (if applicable).**

Before we start with the actual interview, we need you to consent to our research. If you have already signed the informed consent form we sent you, that’s great. If not, we can do this now together.

**Let them know that no personally identifiable data will be recorded and a participant number will be allocated to them**

**Introduction/Background**

What is your position/role within your organisation?

Can you please briefly tell me about your role?

How are you involved in supporting people engaging with self-harm and suicide content?

**Experience of the Online Safety Hub**

How would you describe your experience with using the Online Safety Hub? Probe: Has it improved the experience of interacting/engaging with service users and other services?

What do you feel are the main benefits of using the Online Safety Hub? Probe: Such as guiding your conversation with service users etc

Are there any particularly useful features of the Online Safety Hub? Probe: For example, providing information about…..

Have you had any problems or concerns regarding using the Online Safety Hub? Probe: difficulty in finding time etc

Would you recommend the use of the Online Safety Hub to any other health, social and mental care professionals supporting people engaging in self-harm and suicide content? Why?

**Impact of the Online Safety Hub**

How do you think your interaction with the Online Safety Hub has impacted your ability, if at all, to support people engaging with self-harm and suicide content? Did this have any impact on the conversations you had with the service users? If so how? If not, why?

Has the Online Safety Hub had any impact on your experience/ability to work with other services in supporting people who engage with self-harm and suicide content?

**Implementation Challenges/Opportunities**

What do you think are the factors that will either enable or prevent the successful implementation of the Online Safety Hub across your service?

What are the benefits/challenges to achieving successful implementation of the Online Safety Hub across your service?

What do you think are the main organizational barriers to implementing the Online Safety Hub to support service users? What would help you to overcome them? Prompt: service providers' resistance; difficult to use etc

What kind of information or training, if any, did you undertake to support your learning with the Online Safety Hub? Probe: Is there any further information/training you think should be available for service providers who would like to use the Online Safety Hub?

For you what would successful use of the Online Safety Hub look like?

**Further Development of the Online Safety Hub**

How do you think the Online Safety Hub could be developed or improved further?

Is there anything else that is not currently included in the Online Safety Hub?

**Conclusion**

Do you have anything else you would like to add about the Online Safety Hub? Are there any other issues which you think are relevant in the context of this study?

**Check that they’re ok and there are no safety concerns.**

**Closing and thanks -** check that the participant is still happy for you to use all the information provided and offer the possibility to erase sections of the recording.

Thank them for their time and contribution.

## Topic Guide for Focus Group

*Welcome and introductions, consent and recording*

*Icebreaker: E.g. What’s the weather like today where are you?*

1. Tell us about the kind of role you have, what kind of organisation you work for and what kind of setting (type into chat if the group is very large?)

**Focus:** The aim of the focus group today is to discuss your views and experiences of talking about online harm with people that you support who self-harm or are having suicidal thoughts, and the role of the Samaritans online safety hub in helping you to do that There might be other issues that come up that you would like to talk about, which are relevant or connected, but generally we will ask you to focus on this main topic.

**Taking care of yourself**: you have signed up to this group because you support people with issues of self-harm and suicide. We hope, and expect, that you have support available to you should you be personally affected by this work – and we would hope that the discussion today will not be one that you will find distressing. However, we are aware that these issues are sensitive and can sometimes affect us in unexpected ways and at unexpected times. If for any reason you find that the discussion today starts to cause you distress, please take care of yourself and let us know if you would like us to direct you to any further support after the focus group ends.

**About your own experiences**

1. What experiences have you had of talking to people you support with self-harm and suicide about online harm?

Prompts: have you raised the issue, have they? How has it gone? How has the webinar or the Samaritans’ resource supported you in dealing with the subject?

1. When you are talking to people about online harm, what do you feel most concerned about or unsure about?
   Prompt/listen out for any specific sites or content that they might be concerned about
2. What do you feel most confident about?
   Prompt: Has this changed/improved since taking the online training or accessing the online safety hub
3. If you haven’t spoken to people about online harm, are there any particular reasons why not?

**Experiences with the Online Safety Hub**

1. Tell us about your experiences of using the Samaritans online safety hub – which resources have you used, when and what did you make of them? (Did you miss something?)
2. What do you feel are the main benefits of using the Samaritans online safety hub?
3. Have you had any problems or concerns regarding using the Samaritans online safety hub?
4. What do you think about the content provided (if this has not already come up?) What do you think of the online mode of accessing the resources? Can you think of a better way to use them?
5. How do you think your experience in using the Samaritans online safety hub has impacted/could impact your ability, if at all, to provide support to individuals engaging with self-harm and suicide content?

**Further development of the Online Safety Hub**

1. How do you think the Samaritans online safety hub could be developed or improved further?
2. Is there anything else that is not currently included in the Samaritans online safety hub?
3. Would you recommend the resources to fellow practitioners?
4. What might help or hinder the hub being more widely used to support practitioners?
5. What do you think about your recent participation in the webinar? What would you like in future?

**Thinking about your own experiences and more broadly**

1. How effective do you think talking about online harm can be with people who self-harm or who are suicidal?
2. In what ways might talking to people about online harm help them?
   Prompt: might people use the internet differently as a result?
3. Can you think of any disadvantages of talking to people about online harm?
4. What else is important to consider about this topic?
